# The Cell Death Triggered by the Nuclear Localized RxLR Effector PITG_22798 from *Phytophthora infestans* Is Suppressed by the Effector AVR3b

**DOI:** 10.3390/ijms18020409

**Published:** 2017-02-14

**Authors:** Hongyang Wang, Yajuan Ren, Jing Zhou, Juan Du, Juan Hou, Rui Jiang, Haixia Wang, Zhendong Tian, Conghua Xie

**Affiliations:** 1 Key Laboratory of Potato Biology and Biotechnology (Ministry of Agriculture, China), Huazhong Agricultural University, Wuhan 430070, China; hongyang8318@126.com (H.W.); 13349888011@163.com (Y.R.); zhoujing1018@ sina.cn (J.Z.); karenduren1985@gmail.com (J.D.); houjuanok@163.com (J.H.); jiangrui828@webmail.hzau.edu.cn (R.J.); 13212798446@163.com (H.W.); 2 School of Life Science, Yunnan Normal University, Kunming 650500, China

**Keywords:** cell death, effector, *Nicotiana benthamiana*, *Phytophthora infestans*, plant immunity

## Abstract

Phytopathogenic oomycetes, such as *Phytophthora infestans*, potentially secrete many RxLR effector proteins into plant cells to modulate plant immune responses and promote colonization. However, the molecular mechanisms by which these RxLR effectors suppress plant immune responses are largely unknown. Here we describe an RxLR effector PITG_22798 (Gene accession: XM_002998349) that was upregulated during early infection of potato by *P. infestans*. By employment of agroinfiltration, we observed that *PITG_22798* triggers cell death in *Nicotiana benthamiana*. Confocal microscopic examination showed that PITG_22798-GFP (Green Fluorescent Protein) located in the host nucleus when expressed transiently in *N. benthamiana* leaves. A nuclear localization signal (NLS) domain of PITG_22798 is important for nuclear localization and cell death-inducing activity. Sequence alignment and transient expression showed that *PITG_22798* from diverse *P. infestans* isolates are conserved, and transient expression of *PITG_22798* enhances *P. infestans* colonization of *N. benthamiana* leaves, which suggests that *PITG_22798* contributes to *P. infestans* infection. *PITG_22798*-triggered cell death is dependent on SGT1-mediated signaling and is suppressed by the *P. infestans* avirulence effector 3b (AVR3b). The present research provides a clue for further investigation of how *P. infestans* effector PITG_22798 associates with and modulates host immunity.

## 1. Introduction

Plants are attacked by various pathogens and defend themselves via multiple resistance mechanisms [1,2]. Generally, plant immunity can be divided into two types, pathogen-associated molecular pattern-triggered immunity (PTI) and effector-triggered immunity (ETI) [3]. Fungi and oomycetes can secrete a wide diversity of effectors into host cells to manipulate plant immunity. These effectors are thought to determine the outcome of plant–microbe interactions [4,5].

Two classes of oomycetes effectors target distinct sites in the host plant: apoplastic effectors, such as GIP1, EPI1, and EPICI, are secreted into plant extracellular space, whereas cytoplasmic effectors, such as CRNs (crinkling and necrosis induced protein) and RxLR effectors, are translocated inside the plant cell, where they target different subcellular compartments [6]. CRNs are generally considered as a class of cell death-inducing effectors in plant, such as CRN2 and CRN8 [7], whereas the RxLR type effectors have been shown to manipulate the plant immune network [8,9,10]. RxLR type effectors were classified based on a common sequence pattern identified in oomycete avirulence proteins. They are modular proteins that contain N-terminal signal peptides, RxLR-dEER motif, and diverse and rapidly evolving C-terminal effector domains [11,12]. It is documented that the genomes of *P. infestans*, *P. sojae*, *P. ramorum*, and *Hyaloperonospora arabidopsidis* have more than 550, 390, 370, and 130 genes, respectively, encoding potential effector proteins with RxLR-dEER motifs [8,10]. Some effectors of fungal pathogens may also contain functional variants of the RxLR motif [13]. The major function of RxLR effectors is believed to help pathogens to colonize plants by interacting with critical host targets to suppress plant basic defense responses [1,14,15,16,17]. For example, a *P. infestans* RXLR effector PITG_04314 targets plant PP1c isoforms by re-localizing PP1c isoforms from the nucleolus to the host nucleoplasm to promote late blight disease [18].

Increasing evidence indicates that effectors traffic to a range of subcellular localizations in plant cells and target diverse host proteins to execute their functions [6]. For example, the PITG_04097 nuclear localization is required for both suppression of MAMP (microbe-associated molecular pattern) signaling and virulence function [17]. The *P. infestans* RxLR effector PexRD2 interacts with MAPKKKe in the plant cytosol and specifically inhibits the MAPKKKe-dependent resistance [16]. Many pathogen effector proteins target the nucleus in order to modify host cell physiology, such as CRN8 from *P. infestans* [7].

During transient *Agrobacterium*-mediated expression studies of *P. infestans* RxLR effectors in *Nicotiana benthamiana*, we found that one RxLR-like effector, PITG_22798 (Gene accession: XM_002998349), could induce cell death. In this work, we found that PITG_22798 was localized in the host nucleus and its induction of cell death in *N. benthamiana* required nuclear localization. We also discovered that this induced cell death was dependent on the defense regulator SGT1 (suppressor of G2 allele of skp1) and was suppressed by the RxLR effector, avirulence 3b (AVR3b). The work described herein provides important foundations for further dissection of the roles of *P. infestans* RxLR effector PITG_22798 regulation in plant immunity.

## 2. Results

### 2.1. PITG_22798 Is Induced Early during Infection of P. infestans and Promotes Pathogen Colonization

**Figure 1:**
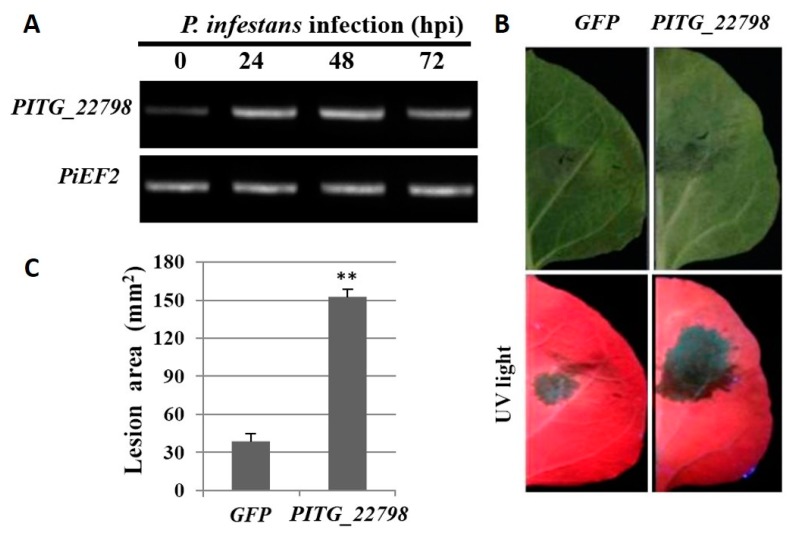
*PITG_22798* is induced during infection of *P. infestans* and promotes colonization. (**A**) Semiquantitative RT-PCR was performed to test *PITG_22798* expression during *P. infestans* infection in potato leaves at 0, 24, 48, 72 h post-inoculation (hpi). The elongation factor 2 gene (*PiEF2*) of *P. infestans* was used as a control to equalize cDNA amounts. (**B**) A typical leaf with larger lesions on the half of the leaf expressing *PITG_22798* in *N. benthamiana*. *Agrobacterium* was used to transiently express *PITG_22798* on one half of a leaf and *GFP* (Green Fluorescent Protein) on the other. Leaves were subsequently infected with *P. infestans*. The pictures were taken at 5 days after inoculation under white light (**top**) and UV light (**bottom**). The OD600 of *Agrobacterium* for transient express of *PITG_22798* is 0.05. (**C**) Lesion area at 5 d after zoospore inoculation following infection of *GFP*- or *PITG_22798*-expressing leaf tissue with *P. infestans* 88069. Results are the mean ± SE of infections from three biological replicates using at least nine leaves each. The asterisk indicates a value significantly different from the *GFP* (*p* < 0.01, *t*-test).

To investigate the expression pattern of *PITG_22798* during *P. infestans* infection, we designed gene-specific primers (Table S1 in Supplementary Material) and performed reverse transcriptase PCR (RT-PCR) using cDNA reverse transcribed from potato leaf RNA isolated at 0, 24, 48, and 72 h after inoculation. The RT-PCR results revealed that *PITG_22798* was upregulated at 24 h and 48 h after inoculation of potato plants with *P. infestans* (Figure 1A). The weak band at 0 h presumably reflects the very lower expression of the *PITG_22798* in *P. infestans* zoospores.

To test whether *PITG_22798* contributes to the virulence of *P. infestans*, we transiently expressed *PITG_22798* in *N. benthamiana* followed by *P. infestans* inoculation. The graph in Figure 1B,C shows an increased *P. infestans* lesion size in the presence of the *PITG_22798* relative to lesions occurring with the *GFP* (Green Fluorescent Protein gene) control, indicating that *PITG_22798* enhances *P. infestans* leaf colonization.

### 2.2. PITG_22798 Causes Cell Death in N. benthamiana

**Figure 2:**
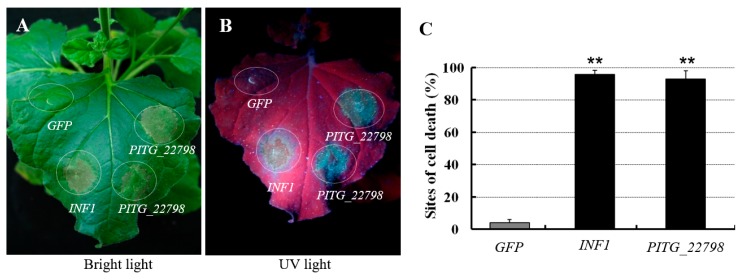
*PITG_22798*-induced cell death in *Nicotiana benthamiana.* Images show cell death induced by *PITG_22798* under white light (**A**) and UV light (**B**). Plant leaves were infiltrated with *A. tumefaciens* cells containing a potato virus X (PVX) vector carrying genes *INF1* (positive control, OD600 = 0.4), *PITG_22798* (OD600 = 0.4), or *GFP* (Green Fluorescent Protein) (negative control, OD600 = 0.4). All pictures were taken at 6 days post-infiltration (dpi). (**C**) The graph shows the percentage of inoculation sites showing cell death. Three *N. benthamiana* plants and four leaves per plant were used for each test. Values are means ± SD. The experiment has been repeated three times with similar results. Asterisks denote values significantly different from *GFP* (*p* < 0.01, *t*-test).

We noted that when we transiently expressed the *PITG_22798* in *N. benthamiana* by agroinfiltration, cell death occurred at 6 days post-infiltration (dpi). The cell death observed was associated with accumulation of autofluorescent compounds. To better visualize the accumulation of such compounds, we examined the inoculated sites under UV light. Similar to *INF1* (positive control), *PITG_22798* resulted in increased autofluorescence in dead and dying cells, whereas GFP (negative control) did not lead to necrosis or autofluorescence in *N. benthamiana* (Figure 2). We also expressed *PITG_22798* in two wild potato species, *S. chacoense* and *S. hjertingii*, by PVX (potato virus X vector)-agroinfection assay, and in *N. tabacum* leaves by agroinfiltration. The results revealed that expression of *PITG_22798* induced cell death in *N. tabacum* but not in potato species (Figure S1), suggesting that *PITG_22798*-induced cell death may not be toxic-like due to the artificially high expression. Overall, the above data suggest that *PITG_22798* could induce cell death in two tested *Nicotiana* species.

### 2.3. PITG_22798 Is Conserved in P. infestans Isolates

**Figure 3:**
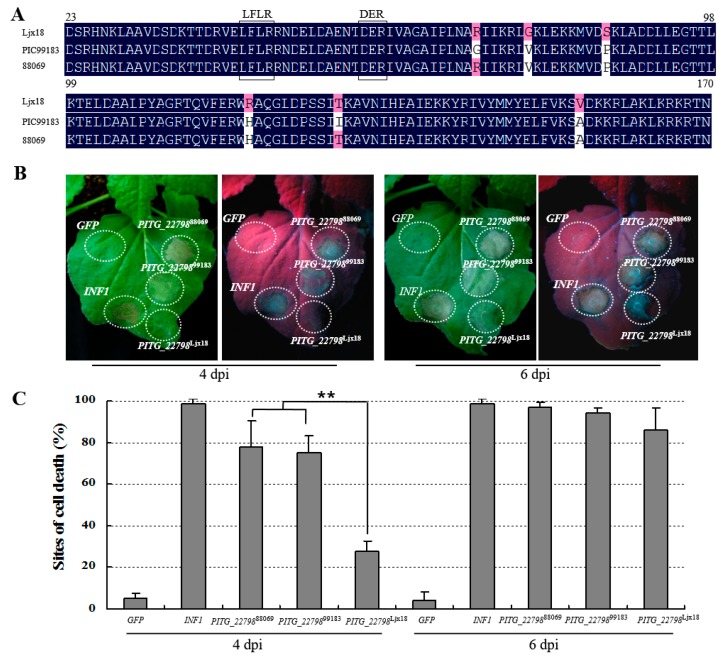
Polymorphisms of PITG_22798 and the cell death induce ability. (**A**) Alignment of PITG_22798 orthologues from 11 *P. infestans* isolates. The amino acid sequences of other 9 orthologues are same as that from 88069. ClustalW was used to make the alignment. Polymorphic amino acids were marked with red. (**B**) *PITG_22798*^Ljx18^ induces a delayed cell death in *N. benthamiana*. *A. tumefaciens* containing *GFP*, *INF1*, *PITG_22798*^88069^, *PITG_22798*^99183^, or *PITG_22798*^Ljx18^ was infiltrated in *N. benthamiana* leaves with an OD600 of about 0.4. *GFP* was used as a negative control while *INF1* was used as a positive control. *PITG_22798*^88069^ and *PITG_22798*^99183^ induce cell death at both 4 dpi and 6 dpi, while *PITG_22798*^Ljx18^ only induces cell death at 6 dpi. (**C**) Graphs show the cell death percentages. Six *N. benthamiana* plants and four leaves per plant were used for each test. Data were collected from 72 infiltration sites based on three independent experiments. Values are means ± SD. Asterisks denote values significantly different from the *PITG_22798*^Ljx18^ (*p* < 0.01, *t*-test). The experiment has been repeated three times with similar results.

*PITG_22798* encodes a protein of 170 amino acids (aa) with a signal peptide from 1–22 aa. It contains the RXLR-like motifs: LFLR-DER. To study the diversity of *PITG_22798*, its orthologues were cloned from 11 diverse *P. infestans* isolates, namely EC1, Ljx18, IPO-C, UK3928A, HB09-23, HB09-16-2, HB09-14-2, HB09-21, HB09-41, 88069, and PIC99183 (Table S2). Among 11 cloned sequences, 9 sequences are identical while the other 2 from Ljx18 and 99183 had only 4 and 2 aa differences, respectively. The alignment of predicted amino acid sequences showed a high conservation with 96% similarity (Figure 3A).

To investigate whether sequence polymorphisms of PITG_22798 affect its ability to trigger cell death, *PITG_22798* orthologues from *P. infestans* isolates 88069 (*PITG_22798*^88069^), 99183 (*PITG_22798*^99183^), and Ljx18 (*PITG_22798*^Ljx18^) were transiently expressed in *N. benthamiana*. The results showed that all of them triggered cell death, while a delayed symptom was observed with the orthologue from Ljx18 (Figure 3B,C).

### 2.4. Effector PITG_22798 Functions in the Nucleus to Induce Cell Death

**Figure 4:**
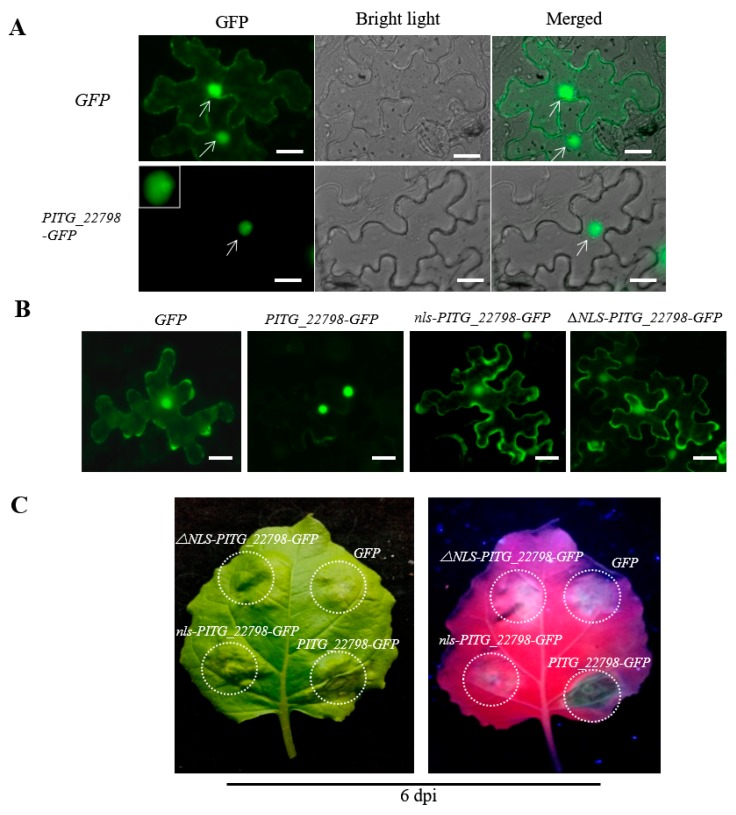
Localization and functional characterization of a nuclear localization signal (NLS) motif for PITG_22798. (**A**) PITG_22798-GFP fusion protein and control GFP were expressed in *N. benthamiana* leaves. Photos show confocal images of epidermal cells at 36 h after agroinfiltration. White arrows indicate the nucleus. PITG_22798-GFP is clearly visible in the nucleus (arrow). Left GFP channel, middle bright field channel, right GFP, and bright field channels merged. The white scale bars represent 20 μm. (**B**) Overview of the mutant construct. *nls-PITG_22798-GFP*, *ΔNLS-PITG_22798-GFP*, *PITG_22798-GFP* and control *GFP* were transiently expressed in *N. benthamiana* leaves using agroinfiltration. Confocal microscopy was used to investigate fusion protein distributions. The epidermal cells transformed by *nls-PITG_22798-GFP* and *ΔNLS-PITG_22798-GFP* showed the same amount of cytosolic fluorescence as the control GFP. Photos show confocal images of mesophyll cells at 36 h after agroinfiltration. The white scale bars represent 20 µm. The OD600 of the *Agrobacterium* culture for localization was 0.01. (**C**) Expression of *PITG_22798* and mutants in *N. benthamiana* leaves by agroinfiltration. The OD600 of the *Agrobacterium* culture for infiltration was 0.4. Photographs of symptoms were taken 6 d after infiltration. Three repeats show similar results. Each repeat includes 24 infiltration sites on 6 *N. benthamiana* plants (4 leaves per plant).

GFP was fused to the C-terminus of PITG_22798 (23–170, without signal peptide) and transiently expressed in *N. benthamiana* by agroinfiltration. To avoid quick cell death, a low-concentration agrobacteria suspension (OD600 = 0.01) was used for agroinfiltration. The results showed that PITG_22798-GFP localizes to the nucleus (Figure 4A). Meanwhile, immunoblots demonstrated that GFP and PITG_22798-GFP fusion proteins were intact (Figure S2), indicating that the fluorescence accurately reflects the localization of PITG_22798. These results strongly suggest that PITG_22798 is a nuclear localized effector.

A predicted nuclear localization signal (NLS) motif (157-KKRLAKLKRKR-167) was located in the C-terminus of PITG_22798. We mutated the NLS of PITG_22798 (nls-PITG_22798) and found that the NLS mutant (23–170, 157-KKRLAKLKAAA-167) and ΔNLS-PITG_22798 (23–170, deleted NLS) mainly accumulated in the cytoplasm rather than nucleus (Figure 4B). It was also observed that the NLS mutant and ΔNLS-PITG_22798 could not induce cell death (Figure 4C), indicating that nuclear localization is required for PITG_22798 to trigger cell death.

**Figure 5:**
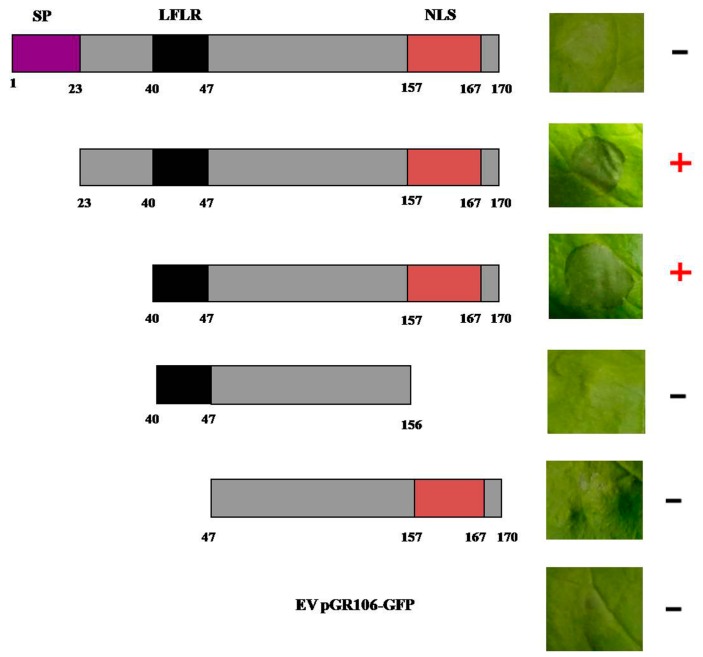
Deletion analysis of PITG_22798. Deletion mutants of *PITG_22798* were transiently expressed in *N. benthamiana* by agroinfiltration. A schematic view of the different mutants and deletion constructs are shown on the **left panel**. Symptoms of infiltration sites are shown on the **right panel**. Plus (+) and minus (−) signs indicate the presence or absence, respectively, of cell death symptoms. The assays were repeated at least three times with similar results. Photograph of symptoms were taken at 6 dpi. SP, signal peptide. LFLR, an RxLR-like motif. NLS, nuclear localization signal sequence.

To further dissect the function of PITG_22798, we employed four deletion constructs of PITG_22798 and tested their ability to induce cell death in *N. benthamiana* (Figure 5). These experiments indicated that a region from 40 to 170 aa of PITG_22798 was necessary for triggering cell death. Deleted LFLR or NLS domain of PITG_22798 lost the ability to induce cell death in *N. benthamiana* (Figure 5). The results further revealed that both the RxLR-like motif (43-LFLR-47) and the predicted C-terminal NLS (157-KKRLAKLKRKR-167) were important domains for cell death induction.

### 2.5. PITG_22798-Mediated Cell Death Requires SGT1, but Not HSP90

**Figure 6:**
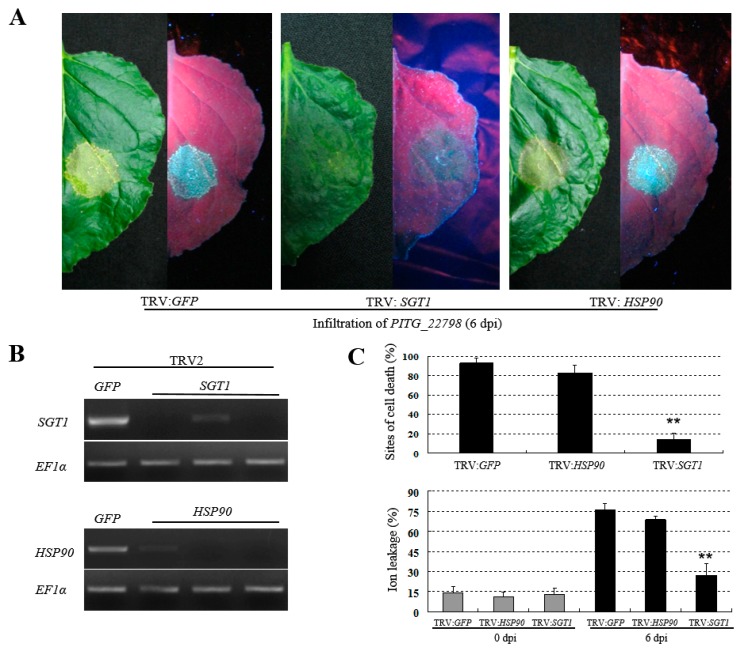
*SGT1* (suppressor of G2 allele of skp1) is required for *PITG_22798*-induced cell death. (**A**) Representative symptoms of *PITG_22798*-induced cell death on *SGT1-* and *HSP90*-silenced *N. benthamiana* leaves at 6 d post-infiltration. (**B**) RT-PCR was done to test the silencing efficiency of *SGT1* and *HSP90*. *EF1α* was used to confirm equal total RNA amounts among samples. (**C**) Quantification of cell death and ion leakage on *SGT1-* and *HSP90*-silenced *N. benthamiana* leaves. Cell death differences were based on three independent experiments scored at 6 dpi (upper panel). Ion leakage was measured at 0 and 6 dpi (lower panel). Results were obtained from 120 infiltration sites based on five independent experiments. Values are means ± SD. Asterisks denote values significantly different from control TRV: *GFP* (*p* < 0.01, *t*-test).

Heat shock protein 90 (HSP90) and SGT1 are considered components of signal-transduction pathways leading to cell death mediated by many NB-LRRs (nucleotide-binding site leucine-rich repeats) cell death. Here, we tested whether SGT1 and HSP90 are required for *PITG_22798*-induced cell death using virus-induced gene silencing (VIGS). Three weeks after infiltration of silencing constructs, newly grown developed leaves were infiltrated with *A. tumefaciens* strains containing *PITG_22798* (Figure 6A). We confirmed that the *SGT1* and *HSP90* transcripts were reduced in the silenced plants compared with negative control infiltrated with tobacco rattle virus (TRV)-*GFP* (Figure 6B). Percentages of cell death were recorded at 6 dpi (days post-infiltration). In *SGT1*-silenced plants, less than 20% of *PITG_22798*-inoculated sites exhibited cell death, whereas over 80% of *GFP*- or *HSP90*-silenced leaves showed cell death (Figure 6C, upper part). This observation was verified by ion leakage analysis, which indicated a significant decline in percentage of total conductivity of *SGT1*-silenced leaves compared with *GFP*- or *HSP90*-silenced leaves (Figure 6C, lower part). These results suggested that cell death triggered by *PITG_22798* in *N. benthamiana* requires the *SGT1*-mediated signaling pathway rather than the *HSP90*-mediated signaling pathway.

### 2.6. AVR3b Suppresses PITG_22798-Triggered Cell Death in N. benthamiana

**Figure 7:**
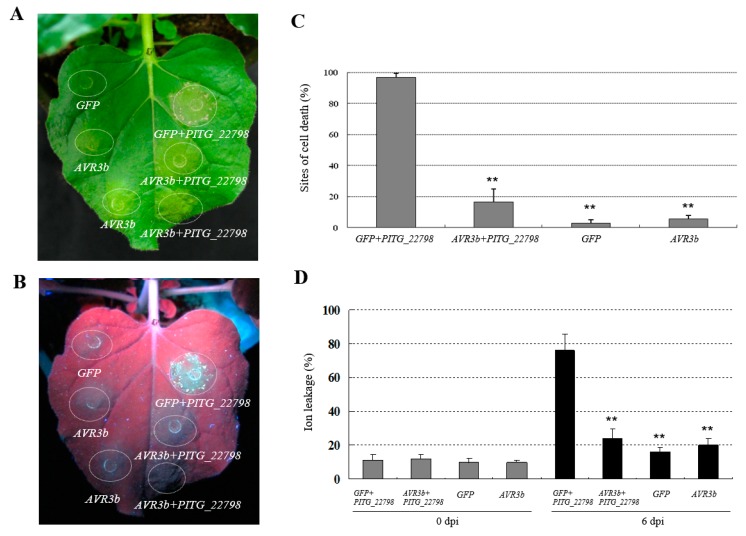
Avirulence 3b (AVR3b) suppresses the cell death induced by *PITG_22798*. *PITG_22798* and *AVR3b* were agro-co-infiltrated in *N. benthamiana* leaves in a 1:1 ratio with a final OD600 of about 0.4. *GFP* and *AVR3b* were used as negative controls. Photos were taken at 6 dpi under normal light (**A**) and UV light (**B**). (**C**) *PITG_22798*-induced cell death is significantly reduced after co-infiltrated with *AVR3b*. Error bars represent SD. Results were obtained from 36 infiltration sites based on three independent experiments. Asterisks indicate significant difference from positive control (*p* < 0.01, *t*-test). (**D**) *PITG_22798*-induced ion leakage is significantly reduced after co-infiltration with *AVR3b*. Error bars represent SD. Asterisks denote values significantly different from positive control (*p* < 0.01, *t*-test). Experiments were repeated three times with similar results.

To study whether other RxLR effectors could interfere with *PITG_22798*-mediated cell death, we selected eight RxLR effectors which were co-agroinfiltrated with *PITG_22798* in *N. benthamiana.* The results obtained at 6 dpi demonstrated that *PITG_22798*-induced cell death was significantly inhibited when it was coexpressed with *AVR3b*, whereas other effectors did not interfere with *PITG_22798*-triggered cell death (Figure S3). Considering percentage of the inoculation sites that showed cell death symptoms and ion leakage by percentage of total conductivity, *AVR3b* significantly suppressed *PITG_22798*-induced cell death (Figure 7). 

## 3. Discussion

Cell death plays a ubiquitous role in plant–microbe interactions and can be associated with both susceptible and resistance responses [19]. However, less is known about RxLR effectors of *P. infestans* that could directly induce cell death in *N. benthamiana*. Until now, only one example has been reported. PexRD2 is such an RxLR effector that can trigger cell death in *N. benthamiana*, and also can interact with host MAPKKK to perturb plant immunity [16,20]. In this work, we identified an RxLR-like effector PITG_22798 which can trigger cell death in *N. benthamiana* (Figure 2) and tobacco. However, expression of *PITG_22798* in potato did not lead to cell death. Cell death is thus unlikely to be associated with the effector function of PITG_22798. Some RxLR effectors have been shown to act as avirulence (AVR) factors, triggering the ETI pathway due to recognition by cognate R proteins [2,12,21]. The cell death triggered by PITG_22798 may result from recognition by an unknown R protein in *N. benthamiana*. This is supported by the fact that PITG_22798-triggered cell death is dependent on SGT1, which is required for many R proteins to function. Even if this is in accordance with the view that *N. benthamiana* possibly possesses a broad-spectrum R protein against *P. infestans* [22], further experiments are needed to support the hypothesis. 

The nucleus is an activity center for both PTI and ETI, and many critical regulators are trafficked there from various subcellular locations following pathogen perception [23,24]. By fusing it to GFP, we showed that PITG_22798 is a nuclear-localized protein (Figure 4A). Moreover, PITG_22798 requires a functional NLS for nuclear accumulation and cell death induction (Figure 4B and Figure 5). Therefore, our results suggest that PITG_22798 may implement its function in the nucleus.

Both SGT1 and HSP90 are critical signaling components required for *R* gene-mediated resistance in several plant species against various plant pathogens, including fungi and bacteria [25]. These proteins also play a role in stabilization of R proteins [26,27,28]. Additionally, non-RxLR effectors INF1- or INF2A-mediated cell death were also suppressed when *NbSGT1* was silenced in *N. benthamiana*, indicating that SGT1 function is not only limited to the NB-LRR proteins but also required for the immune response that is triggered by non-NB-LRR-type proteins [26,29]. Our findings demonstrated that SGT1, rather than HSP90, is required for PITG_22798-triggered cell death in *N. benthamiana* (Figure 6). This finding is similar to the report that AVR-blb2, another *P. infestans* RxLR effector, triggered cell death, which needs SGT1 rather than HSP90 in the presence of the cognate Rpi-blb2 protein [28]. SGT1 was reported to act as an adaptor of plant NLR (nucleotide-binding domain- and leucine-rich repeat-containing) proteins (including R proteins) and protect them from degradation [27]. Although the question of how SGT1 regulates PITG_22798 cell death induction could not be addressed, we hypothesize that NbSGT1 may be required for a cognate R protein that recognizes PITG_22798 in *N. benthamiana*. 

Hundreds of predicted RxLR effector-encoding genes exist in the genomes of sequenced oomycete plant pathogens. Surveys of *Phytophthora* RxLR effectors identified many that could suppress cell death and a few that could induce cell death, such as cell death inducers Avh147, Avh238, and Avh241 [30,31]. Many of the suppressors, such as AVR3a^KI^, AVR3b, and PITG_02680 of *P. infestans*, and Avr1b and Avh331 of *P. sojae*, can suppress cell death activated by PTI [17,18,32,33]. Here, we showed that AVR3b can suppress the cell death triggered by PITG_22798 (Figure 7 and Figure S3). It has been reported that AVR3b, as an avirulence factor, can trigger cell death due to recognition by the cognate R3b protein [34]. It also, as a virulence factor, can suppress INF1-triggered cell death and promote *P. infestans* infection in *N. benthamiana* [17]. AVR3a and AVR3b can all suppress INF1-triggered cell death [17]. However, only AVR3b can suppress PITG_22798-induced cell death. Therefore, it is possible that PITG_22798 cell death is a different pathway to INF1 cell death. It is also possible that AVR3b has more than one virulence function. It remains unclear, however, whether AVR3b directly blocks recognition of PITG_22798, or impacts on the expression, import, and stability of PITG_22798, or by another mode that may associate with different subcellular localization of PITG_22798 (nucleus) and AVR3b (cytosol and plasma membrane). A possible explanation could be that AVR3b may interfere with PITG_22798 through an intermediary signaling pathway that is required for PITG_22798 to trigger cell death, which merits further investigation. Further studies are needed to clarify the molecular mechanism underlying how AVR3b suppresses PITG_22798-mediated cell death. However, the fact that it is able to suppress this cell death explains why *N. benthamiana* is not resistant to *P. infestans*, and also why PITG_22798 is able to enhance *P. infestans* colonization (Figure 1B,C), which is likely associated with its virulence function.

## 4. Materials and Methods

### 4.1. Microbial Strains, Plants, and Culture Conditions

*Escherichia coli* DH5α was cultured at 37 °C in LB (Luria-Bertani) medium and used for cloning and propagation of recombinant plasmids. *Agrobacterium tumefaciens* strain GV3101 used for transient expression was cultured at 28 °C in YEB (Yeast Extract Broth) medium using appropriate antibiotics. *P. infestans* isolates used in this study are shown in Supplementary Material, Table S2. All isolates were cultured on Rye agar medium at 22 °C. Plants of *N. benthamiana*, *N. tabacum*, *S. chacoense*, *S. hjertingii*, and a Chinese potato variety “E-potato 3” (*S. tuberosum* cv.) were grown at 25 °C in greenhouse under 16/8 h light/dark photoperiod. 

### 4.2. Construction of Plasmids

The deletion mutants were obtained by PCR amplifications using appropriate primers (Table S1). For protein fusions, PITG_22798 (23–170 aa, without signal peptide) was cloned from plasmid containing PITG_22798 using Gateway technology (Invitrogen, Carlsbad, CA, USA) with specific primers shown in Table S1. *PITG_22798* was inserted into pK7FWG2 vector to produce C-terminal GFP fusion vector, which was then transformed into the *A. tumefaciens* strain GV3101.

To clarify whether other RxLR effectors could interfere with PITG_22798-triggered cell death, eight RxLR effector genes were cloned and constructed into pGR106 vector (Table S3). Eight RxLR effectors of *P. infestans* included AVR3a^KI^ [35], AVR2 [36], AVR3b [34], PITG_21388 [37], PITG_14783, PITG_20303, and PITG_13959 [17], and PITG_23008 [10]. Primers used for plasmid construction and other constructs used in this study are documented in the Table S1. All above plasmids were validated by sequencing in Shanghai Sunny Biotechnology Co., Ltd. (Shanghai, China). 

### 4.3. Expression Analysis of PITG_22798 and P. infestans Infection Assay

Detached leaves of potato (“E-potato-3”) of 8-week-old plants were inoculated with 10 µL zoospores from *P. infestans* isolate HB0914-2 at 5 × 10^4^ mL^−1^ using the method of Vleeshouwers et al. [38]. Leaf discs (1 cm diameter) of equal sizes surrounding the inoculation sites were collected at 0, 24, 48, and 72 h after inoculation and frozen in liquid nitrogen for immediate use or stored at −80 °C for RNA extraction. The RNA extraction and cDNA synthesis were done as previously described [39]. Reverse transcriptase PCR (RT-PCR) was used to examine *PITG_22798* expression at different time points. The constitutively expressed *PiEF2* (Gene accession: XM_002901697.1) was used as a reference for equalizing cDNA amounts in each reaction. The RT-PCR primers used are listed in Table S1.

*Agrobacterium tumefaciens* transient assays (ATTA) in combination with *P. infestans* infection were carried out as described [15]. Briefly, *Agrobacterium* cultures were resuspended in agroinfiltration medium at a final concentration of OD600 = 0.05 and used for transient expression in *N. benthamiana* by agroinfiltration. After 1 day, each infiltration site was inoculated with 10 µL zoospores from *P. infestans* isolate 88069 at 5 × 10^4^ mL^−1^. Lesions were measured and photographed at 5 days postinfection and data was collected from three biological replicates (each replicate with at least nine leaves). 

### 4.4. Cloning and Sequence Analysis of PITG_22798

For cloning of *PITG_22798*, *P. infestans* genomic DNA was used as templates. *P. infestans* mycelia were scraped from the rye agar medium surface after cultured for about 14 d for DNA isolation using the DNA isolation kit (Sangon Biotech, Shanghai, China). High-fidelity *Pfu* polymerase (Vazyme Biotech Co, Ltd., Nanjing, China) was used for amplification of *PITG_22798* orthologues (without signal peptide) and the primers are shown in Table S1. The PCR products were digested with both *Cla*I and *Not*I and ligated into a binary vector pGR106 vector (potato virus X vector), which was then transformed into *A. tumefaciens* strain GV3101 for *Agrobacterium*-mediated transient expression as described by Du et al. [40]. All the above plasmids were validated by sequencing in Shanghai Sunny Biotechnology Co., Ltd. (Shanghai, China). 

The sequence analysis of *PITG_22798* was conducted with ClustalW (http://clustalw.ddbj.nig. ac.jp/index.php?lang=ja) to create multiple sequence alignments, which were then manually adjusted to minimize the number of implied mutations. SignalP 4.1 server (http://www.cbs.dtu.dk/ services/SignalP/) was used for discriminating signal peptides. Sequence translation was done at http://web.expasy.org/translate/. NLS sequence domain of PITG_22798 was predicted by NLStradamus with a prediction cutoff value of 0.6. NLStradamus uses hidden Markov models (HMMs) (http://www.moseslab.csb.utoronto.ca/NLStradamus/) to predict novel NLSs in proteins [41].

### 4.5. Agroinfiltration and PVX-Agroinfection Assay

Agroinfiltration in *N. benthamiana* and PVX (potato virus X vector)-agroinfection in potato were performed as described elsewhere [40]. At least six plants were agroinfiltrated for each treatment and four apical expanded leaves were used per plant. The infiltrated plants were maintained in plant growth chambers at 22 °C with a 16/8 h light/dark photoperiod.

To check the different induction of *PITG_22798* homologs in *N. benthamiana* plants, three independent tests were conducted, each contained 24 leaves of 6 plants. The cell death was measured at 4 and 6 dpi and *t*-test was used for comparison with *PITG_22798*^Ljx18^. The number of the positive cell deaths (i.e., more than 50% of the inoculated region produces clear cell death) were counted as described previously [36] and expressed as the mean percentage of total inoculations sites.

To look into the effects of other RxLR type effectors on *PITG_22798*-induced cell death in *N. benthamiana* leaves, *A. tumefaciens* cells carrying the cell death-inducing gene (*PITG_22798*) and each other RxLR effector genes were mixed in a 1:1 ratio and then infiltrated by following the method mentioned above. Long-wave UV lamp (Black Ray, B-100A, San Gabriel, CA, USA) was used to monitor the autofluorescence of dead cells where phenolic compounds were released. 

### 4.6. Western Blot and Confocal Microscopic Examination

*Nicotiana benthamiana* leaves infiltrated with *A. tumefaciens* containing *GFP* (Green Fluorescent Protein gene) and *PITG_22798-GFP* were harvested at 36 h post-inoculation (hpi). The stability of GFP fusion protein was tested by Western blot as previously described [20]. Briefly, 100 mg leaf tissue was put into 1 mL protein RIPA lysis buffer. After centrifugation, the protein supernatant was suspended in 10 µL 2× SDS loading buffer, and then loaded onto a 12% SDS-PAGE gel for fractionation. After electrophoresis, gel was blotted onto a PVDF membrane for 1.5 h at 30 V. The membrane was blocked using TBS plus 4% nonfat dry milk (TBSM) for 2 h, followed by addition of the monoclonal GFP antibody (Sigma-Aldrich, St. Louis, MO, USA) at 1:3000 dilution and incubated at room temperature. After 1 h, the membrane was washed with TBS containing 0.05% Tween-20 (TBST) five times, and then the secondary antibody was added at 1:8000 dilution for 1 h at room temperature, followed by five washes. Protein gel-blotting chemiluminescence detection kit (Thermo Scientific Pierce, Rockford, IL, USA) was used according to the manufacturer’s instructions to develop the blots on light-sensitive autoradiograph films.

Subcellular localization of PITG_22798 was detected by confocal microscopy *A. tumefaciens* (OD600 = 0.01) containing GFP was pressure infiltrated in the left half of a leaf of 4-week-old *N. benthamiana*, while *PITG_22798* was infiltrated in the right half. The infiltrated cells were observed using a LSM510 Meta confocal microscope (Carl Zeiss, Jena, Germany) at 36 hpi. The excitation wavelengths used for GFP was 488 nm.

### 4.7. Virus-Induced Gene-Silencing (VIGS) Assay

VIGS was performed as previously described [42,43]. TRV-*PDS* was used as a positive control and TRV-*GFP* was used as a negative control. At four-leaf stage, *N. benthamiana* leaves were infiltrated by *A. tumefaciens* containing TRV-*GFP*, TRV-*PDS*, TRV-*SGT1*, and TRV-*HSP90*. Three weeks later, when positive control leaves became totally white, RNA was extracted from the leaves using Trizol Reagent (Vazyme Biotech Co, Ltd., Nanjing, China) according to the manufacturer recommendations. Primers RT-*NbSGT1* and RT-*NbHSP90* were used for RT-PCR (Table S1). *EF-1α* was used as a control for equalizing cDNA amounts in each reaction. 

### 4.8. Measurement of the Ion Leakage

Ion leakage was measured as previously described [44]. After infiltration, leaf discs (1 cm diameter) were rinsed three times (2–3 min per time) with distilled water and subsequently floated on 10 mL of distilled water. The electrolyte leakage in the solution was measured after 3 h of floating at room temperature using a conductivity meter (METTLER TOLEDO FE30, Zurich, Switzerland). Total conductivity was obtained after keeping the flasks in an oven (90 °C) for 30 min. Results were shown as percentage of total conductivity.

## 5. Conclusions

In summary, we report a nuclear-localized *P. infestans* RxLR effector PITG_22798 which could trigger cell death in *N. benthamiana* and enhance *P. infestans* colonization. PITG_22798-triggered cell death requires its nuclear localization and can be suppressed by the *P. infestans* effector AVR3b. PITG_22798-triggered cell death is dependent on the SGT1-mediated signaling pathway. Although the exact mechanisms of signaling crosstalk remain unclear, our results provide useful information for in-depth investigation of how PITG_22798 modulates defense of *N. benthamiana*. The next challenge is to better understand the role of PITG_22798 and its targets in the progression of disease in important host crop potato. Up to now, we have identified several interacting proteins in potato, which will help us further investigate its function involved in manipulating of potato immunity.

## Supplementary Materials

Supplementary materials can be found at www.mdpi.com/1422-0067/18/2/409/s1.

## Abbreviations

PTI	Pathogen-associated molecular pattern triggered immunity
ETI	Effector-triggered immunity
RT-PCR	Reverse transcriptase PCR
VIGS	Virus-induced gene silencing
NLS	Nuclear localization signal
hpi	Hours post inoculation
dpi	Days post infiltration
