# Supplementary material for: The Cell Death Triggered by the Nuclear Localized RxLR Effector PITG_22798 from Phytophthora infestans Is Suppressed by the Effector AVR3b
Source: Int J Mol Sci. 2017 Feb 14;18(2):409. doi: 10.3390/ijms18020409 (PMC5343943; doi:10.3390/ijms18020409)
Supplement: Supplementary file 1 [file ijms-18-00409-s001.pdf]

Supplementary Materials: The Cell Death Triggered by the Nuclear Localized RxLR Effector PITG_22798 from *Phytophthora infestans* Is Suppressed by the Effector AVR3b

Hongyang Wang, Yajuan Ren, Jing Zhou, Juan Du, Juan Hou, Rui Jiang, Haixia Wang, Zhendong Tian and Conghua Xie

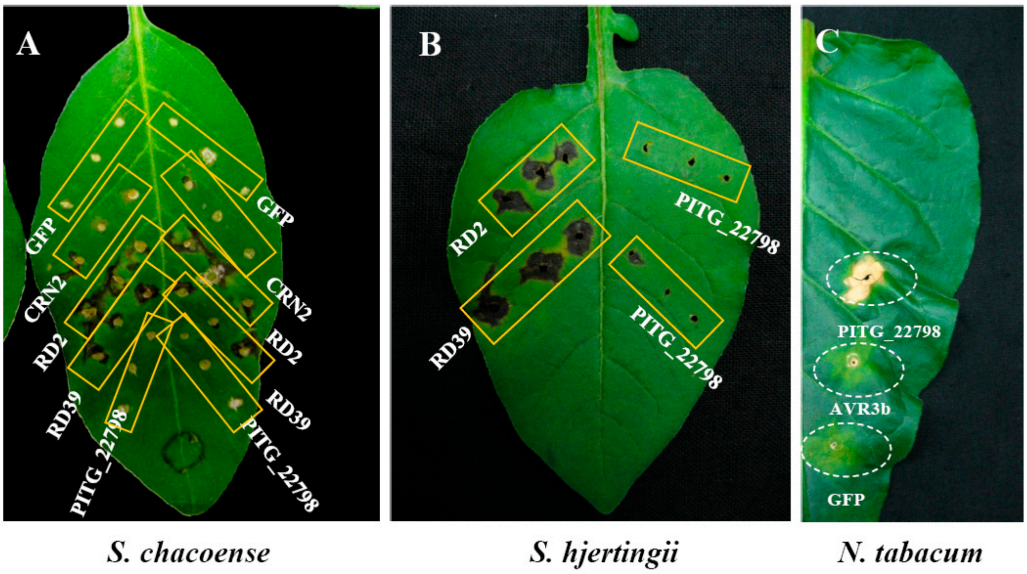

Figure S1. Functional screening of *P. infestans* effectors in wild potato species *S. chacoense*, *S. hjertingii*, and *N. tabacum*. *Agrobacterium* clones expressing *RD2*, *RD39*, and *PITG_22798* of *P. infestans* were tooth-pick inoculated in leaves of *S. chacoense* (**A**) and *S. hjertingii* (**B**). CRN2 (crinkling and necrosis induced protein gene 2) was used as a positive control and *GFP* (green fluorescent protein gene) was used as a negative control. Two weeks after inoculation, the expanding cell death was photographed. (**C**) Leaves of *N. tabacum* were infiltrated with *A. tumefaciens* carrying *PITG_22798*, *AVR3b*, and *GFP* (negative control). Photographs were taken 12 dpi.

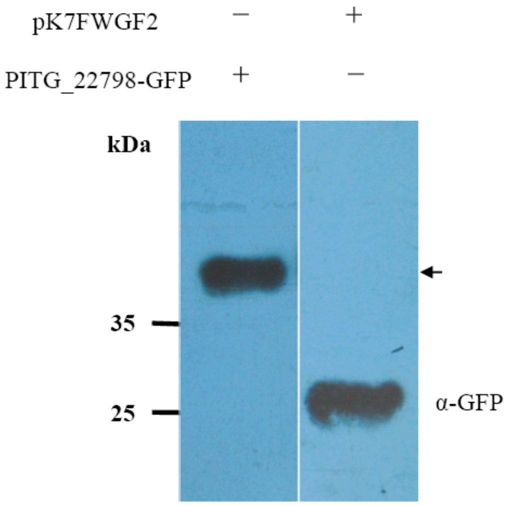

Figure S2. Western blot analysis of proteins PITG_22798-GFP and GFP-empty. Immunoblots show the stability of PITG_22798-GFP. Arrows indicated the expected size (43 kDa). Plus (+) and minus (−) signs indicate the presence or absence respectively.

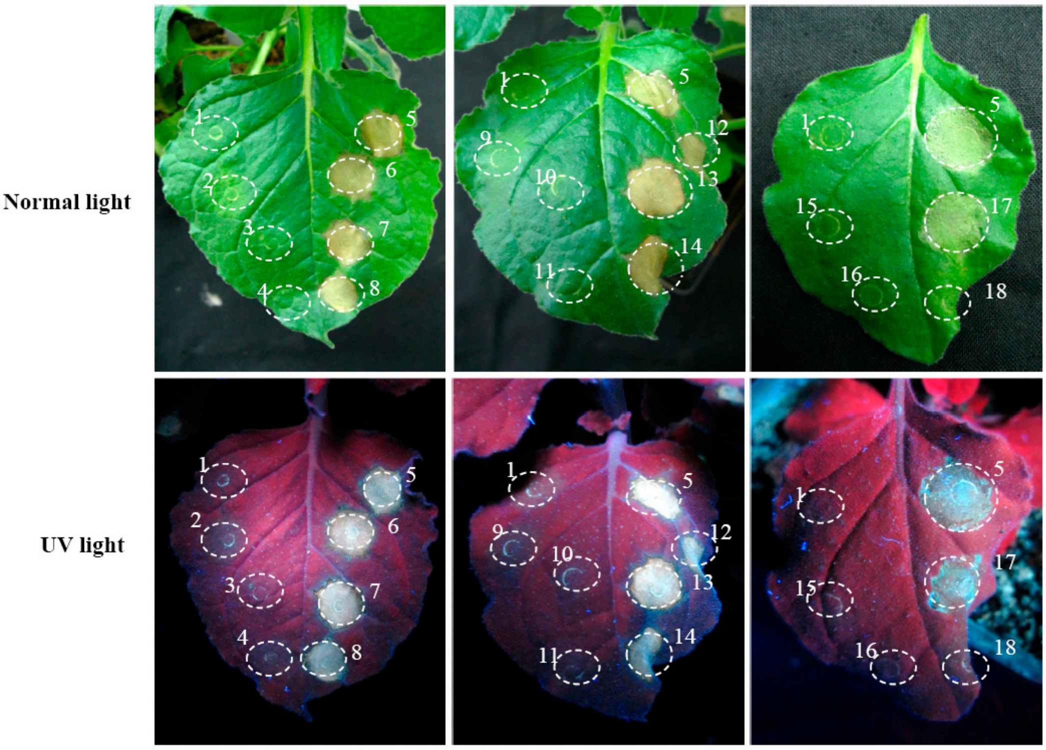

Figure S3. Test of effectors on the suppression of *PITG_22798*-induced cell death. *PITG_22798* was co-agroinfiltrated with the following effectors or controls in a 1:1 ratio with a final OD600 of about 0.4: (1) *GFP*; (2) *AVR2*; (3) *PITG_21388*; (4) *PITG_14783*; (5) *GFP+PITG_22798*; (6) *AVR2* + *PITG_22798*; (7) *PITG_21388* + *PITG_22798*; (8) *PITG_14783* + *PITG_22798*; (9) *PITG_20303*; (10) *PITG_13959*; (11) *PITG_23008*; (12) *PITG_20303* + *PITG_22798*; (13) *PITG_13959* + *PITG_22798*; (14) *PITG_23008* + *PITG_22798*; (15) $AVR3a^{KI}$; (16) *AVR3b*; (17) $AVR3a^{KI}$ + *PITG_22798*; (18) *AVR3b* +*PITG_22798*. Pictures were taken at 7 dpi under normal light and UV light.

Table S1. Primers used in vectors construction, gene cloning, and PCR.

Primer Name	Gene Accession No.	Sequence (5′–3′)	Corresponding Plasmids
PITG_22798$^{1-170}$-F	XM_002998349	GCGATCGATATGAGATGCTACTACGTCCTTA	pGR106-*PITG_22798*$^{1-170}$
PITG_22798$^{1-170}$-R		GATGCGGCCGCTTAATCGTTGGTCCTCTTCCTTT	
PITG_22798$^{23-170}$-F		GCGATCGATATGGACTCAAGGCACAACAAGTTAGC	pGR106-*PITG_22798*$^{23-170}$
PITG_22798$^{23-170}$-R		GATGCGGCCGCTTAATCGTTGGTCCTCTTCCTTT	
PITG_22798$^{40-170}$-F		GCATCGATATGAGAGTGGAGCTATTTCTGCGTCGGA	pGR106-*PITG_22798*$^{40-170}$
PITG_22798$^{40-170}$-R		GATGCGGCCGCTTAATCGTTGGTCCTCTTCCTTT	
PITG_22798$^{40-156}$-F		GCATCGATATGAGAGTGGAGCTATTTCTGCGTCGGA	pGR106-*PITG_22798*$^{40-156}$
PITG_22798$^{40-156}$-R		GATGCGGCCGCATCAGCAGACTTTACGAAGAGCTCG	
PITG_22798$^{47-170}$-F		GCATCGATATGCGGAATGATGAATTGGACGCTG	pGR106-*PITG_22798*$^{47-170}$
PITG_22798$^{47-170}$-R		GATGCGGCCGCTTAATCGTTGGTCCTCTTCCTTT	
PITG_22798-GFP-F		AAAAAGCAGGCTTCACCATGGACTCAAGGCACAACAAGTT	pK7FWG2-*PITG_22798*
PITG_22798-GFP-R		AGAAAGCTGGGTCTTAATCGTTGGTCCTCTTCC	
ΔNLS-PITG_22798-GFP-F		AAAAAGCAGGCTTCACCATGGACTCAAGGCACAACAAGTT	pK7FWG2-ΔNLS-*PITG_22798*
ΔNLS-PITG_22798-GFP-R		AGAAAGCTGGGTCTTAATCGTTATCAGCAGACTT	
nls-PITG_22798-GFP-F		AAAAAGCAGGCTTCACCATGGACTCAAGGCACAACAAGTT	pB7WGF2-*PITG_22798*^{165A166A167A}
nls-PITG_22798-GFP-R		AGAAAGCTGGGTCTTAATCGTTGGTTGCTGCTGCTTTTAGCTT	
AVR3b-F	XM_002997802	ATCCCGGGATGACGTACTCGACTTCAAAGGGGGA	pGR106-*AVR3b*
AVR3b-R		GATGCGGCCGCTTAGAAATTGTTCTTTGCGGTCA	
PITG_23008-F	XM_002899560	GCATCGATATGAATTCTGCGGTTGCGGGCAAG	pGR106-*PITG_23008*
PITG_23008-R		GATGCGGCCGCTTATTTATAACCCAGTCTCATT	

Table S1. *Cont.*

Primer Name	Gene Accession No.	Sequence (5′–3′)	Corresponding Plasmids
PITG_21388-F	KF154438	CGCATCGATGGTTTCATCCAATCTCAACACCGCCG	pGR106-*PITG_21388*
PITG_21388-R		GATGCGGCCGCTATACGATGTCATAGCATGACA	
TRV-NbSGT1-F	AF494083	GCGAATTCCGAACAAGGCCATTGAGTTA	TRV-*NbSGT1*
TRV-NbSGT1-R		GATGGATCCCTCCTCTGGCTTCTGGTAAA	
TRV-NbHSP90-F	AY368904	GCGAATTCTGTCTGGGAATCTCAAGC	TRV-*NbHSP90*
TRV-NbHSP90-R		GATGGATCCTTCGTCAACCTCCTCTAC	
RT-PITG_22798-F		GACTCAAGGCACAACAAGTTAGC	
RT-PITG_22798-R		TTAATCGTTGGTCCTCTTCCTTT	
RT-NbSGT1-F	AF494083	TCGCCGTTGACCTGTACACTCA	
RT-NbSGT1-R		GCAGGTGTTATCTTGCCAAACA	
RT-NbHSP90-F	AY368904	ATGATTGGGCAATTTGGT	
RT-NbHSP90-R		ACACGACGCACATACAGC	
Nbef-1α-F	AY206004	CCAAGCTGACTGTGCTGTCC	
Nbef-1α-R		AAGCAAGCAATGCGTGCTC	
PiEF2-F	XM_002901697	TGACGCTATCGCCAAGGAATC	
PiEF2-R		TAACGCTGAGCCGTAATGGGGG	

Underline represent restriction sites, *Cla*I site (ATCGAT), *Sma*I site (CCCGGG), *Not*I site (GCGGCCGC), *BamH*I site (GGATCC), and *EcoR*I site (GAATTC).

Table S2. *Phytophthorainfestan*s isolates used in this study.

Isolate	Virulence Spectra	Reference
EC1_DC2005	1.3.4.7.10.11	[1]
Ljx18	3.4.7.10.11	[2]
IPO-C	1.2.3.4.5.6.7.10.11	[3]
UK3928A	1.2.3.4.5.6.7.9.10.11	[4]
88069	1.3.4.7	[5]
PIC99183	1.3.4.5.7.8.10.11	[6]
HB09-41	1.2.3.4.5.6.7.8.9.10.11	Unpublished data
HB09-21	1.2.3.4.5.6.7.8.9.10.11	Unpublished data
HB09-23	1.2.3.4.5.6.7.8.9.10.11	Unpublished data
HB09-16-2	1.2.3.4.5.6.7.8.9.10.11	Unpublished data
HB09-14-2	1.2.3.4.5.6.7.8.9.10.11	[2]

Table S3. The RxLR effectors used in this study.

No.	RxLR Gene	*P. infestans* Isolate for Cloning Corresponding Gene
1	*AVR2*	88069
2	*AVR3a*^{KI}	88069
3	*AVR3b*	PIC99183
4	*PITG_21388*	HB09-14-2
5	*PITG_14783*	HB09-14-2
6	*PITG_20303*	HB09-14-2
7	*PITG_23008*	88069
8	*PITG_13959*	HB09-14-2
9	*PITG_22798*	88069
10	*PITG_22798*	Ljx18
11	*PITG_22798*	99183

The RxLR effectors were selected and cloned into the pGR106 vector according to previous studies [7,8].

Reference

1. Armstrong, M.R.; Whisson, S.C.; Pritchard, L.; Bos, J.I.; Venter, E.; Avrova, A.O.; Rehmany, A.P.; Böhme, U.; Brooks, K.; Cherevach, I.; et al. An ancestral oomycete locus contains late blight avirulence gene *Avr3a*, encoding a protein that is recognized in the host cytoplasm. *Proc. Natl. Acad. Sci. USA.* **2005**, *102*, 7766–7771.
2. Wang, H.; Sun, C.; Jiang, R.; He, Q.; Yang, Y.; Tian, Z.; Tian, Z.; Xie, C. The dihydrolipoyl acyltransferase gene *BCE2* participates in basal resistance against *Phytophthora infestans* in potato and *Nicotiana benthamiana*. *J. Plant Physiol.* **2014**, *171*, 907–914.
3. Jo, K.R.; Arens, M.; Kim, T.Y.; Jongsma, M.A.; Visser, R.G.F.; Jacobsen, E.; Vossen, J.H. Mapping of the *S. demissum* late blight resistance gene *R8* to a new locus on chromosome IX. *Theor. Appl. Genet*. **2011**, *123*, 1331–1340.
4. Cooke, D.E.; Cano, L.M.; Raffaele, S.; Bain, R.A.; Cooke, L.R.; Etherington, G.J.; Deahl, K.L.; Farrer, R.A.; Gilroy, E.M.; Goss, E.M.; et al. Genome analyses of an aggressive and invasive lineage of the Irish potato famine pathogen. *PLoS Pathog.* **2012**, 8, e1002940.
5. Van West, P.; de Jong, A.J.; Judelson, H.S.; Emons, A.M.; Govers, F. The *ipiO* gene of *Phytophthora infestans* is highly expressed in invading hyphae during infection. *Fungal Genet. Biol*. **1998**, *23*, 126–138.
6. Flier, W.G.; Grünwald, N.J.; Kroon, L.P.; Van Den Bosch, T.B.; Garay-Serrano, E.; Lozoya-Saldana, H.; Bonants, P.; Turkensteen, L.J. *Phytophthora ipomoeae* sp. nov., a new homothallic species causing leaf blight on *Ipomoea longipedunculata* in the Toluca Valley of central Mexico. *Mycol. Res*. **2002**, *106,* 848–856.
7. Oh, S.K.; Young, C.; Lee, M.; Oliva, R.; Bozkurt, T.O.; Cano, L.M.; Win, J.; Bos, J.I.; Liu, H.Y.; van Damme, M.; et al. In planta expression screens of *Phytophthora infestans* RXLR effectors reveal diverse phenotypes, including activation of the *Solanum bulbocastanum* disease resistance protein Rpi-blb2. *Plant Cell* **2009**, *21*, 2928–2947.
8. Haas, B.J.; Kamoun, S.; Zody, M.C.; Jiang, R.H.; Handsaker, R.E.; Cano, L.M.; Grabherr, M.; Kodira, C.D.; Raffaele, S.; Torto-Alalibo, T.; et al. Genome sequence and analysis of the Irish potato famine pathogen *Phytophthora infestans*. *Nature* **2009**, *461*, 393–398.
